# Enhancement of Light Extraction Efficiency Using Wavy-Patterned PDMS Substrates

**DOI:** 10.3390/nano15030198

**Published:** 2025-01-27

**Authors:** Jian Cheng Bi, Kyo-Cheol Kang, Jun-Young Park, Junbeom Song, Ji-Sung Lee, Hyejung Lim, Young Wook Park, Byeong-Kwon Ju

**Affiliations:** 1 Display and Nanosensor Laboratory, Department of Electrical Engineering, Korea University, Seoul 02841, Republic of Korea; vlfrkatjd@korea.ac.kr (J.C.B.); kgc430@korea.ac.kr (K.-C.K.); mrjoon123@korea.ac.kr (J.-Y.P.); jnh04107@korea.ac.kr (J.S.); js103412@korea.ac.kr (J.-S.L.); hyejung07@korea.ac.kr (H.L.); 2 Department of Semiconductor and Display Engineering, Sun Moon University, Asan-si 31460, Republic of Korea

**Keywords:** polydimethylsiloxane (PDMS), O_2_ plasma, organic light-emitting diodes (OLEDs), light extraction, viewing angle characteristics, surface plasmon polaritons (SPPs)

## Abstract

This study introduces an organic light-emitting diode (OLED) light extraction method using a wavy-patterned polydimethylsiloxane (PDMS) substrate created via oxygen (O_2_) plasma treatment. A rapid fabrication process adjusted the flow, pressure, duration, and power of the O_2_ plasma treatment to replicate the desired wavy structure. This method allowed the treated samples to maintain over 90% total transmittance and enabled controlled haze adjustments from 10% to 70%. Finite-difference time-domain (FDTD) simulations were employed to determine optimal amplitudes and periods for the wavy structure to maximize optical performance. Further experiments demonstrated that bottom-emitting green fluorescent OLEDs constructed on these substrates achieved an external quantum efficiency (EQE) of 3.5%, representing a 97% improvement compared to planar PDMS OLEDs. Additionally, color purity variation was minimized to 0.044, and the peak wavelength shift was limited to 10 nm, ensuring consistent color purity and intensity even at wide viewing angles. This study demonstrates the potential of this cost-effective and efficient method in advancing high-quality display.

## 1. Introduction

Organic light-emitting diodes (OLEDs) are attracting considerable interest as the next generation of display technology owing to their superior properties such as high contrast ratios, rapid response times, low energy consumption, wide viewing angles, and compatibility with devices with various form factors [1,2,3,4]. However, the broader adoption of the OLED technology faces significant hurdles, including issues with narrow viewing angles and inefficient light outcoupling [5,6,7].

The efficiency of OLEDs is characterized by the external quantum efficiency (η_ext,_ EQE), which can be expressed as follows:

(1) $$\eta_{ext}=\eta_{int}\times \eta_{oc}=\gamma \times \eta_{rad.eff}\times \eta_{\gamma }\times \eta_{oc},$$

where γ is the charge balance ratio, η_γ_ is the radiative spin state, and η_rad.eff_ is the radiative efficiency of the emitter [8]. These parameters collectively define the internal quantum efficiency (η_int_), representing the ratio of electron and hole recombination at each electrode that produces excitons in the emission layer (EML) of the OLEDs. By multiplying η_int_ with the outcoupling efficiency (η_oc_), η_ext_ can be calculated. η_oc_ represents the fraction of photons emitted from the OLEDs. Despite advancements in materials pushing η_int_ close to 100%, η_ext_ remains capped at 20–30% when emitted into air, primarily owing to the high refractive index contrast at the interface between two layers. According to Snell’s law, this contrast creates a small critical angle, leading to increased total internal reflection (TIR) and reduced optical transmission [9,10,11]. OLEDs consist of multiple thin films, creating numerous interfaces where light interactions occur. These include the interface between air (n = 1) and glass (n = 1.5), known as the substrate mode, and between glass and ITO/organic layers (n = 1.8–2.2), referred to as the waveguide mode. Additionally, the surface plasmon polariton (SPP) mode arises from the dielectric mismatch between a metal electrode and an organic material. 

To address the challenges associated with light extraction, numerous studies have explored various techniques. These include the use of micro lens arrays [12,13,14], the fabrication of nanostructures through surface texturing [15,16,17], incorporation of scattering layers using nanoparticles or nanorods in polymer thin films [18,19,20], and deployment of periodic grids to isolate specific light modes [21,22,23]. Lin, Bo-Yen et al. utilized blazed gratings to fabricate polydimethylsiloxane (PDMS) molds, creating Polymethyl methacrylate-based corrugated structures that served as internal light extraction layers, achieving a 33.33% improvement in EQE [24,25,26]. Similarly, Bae, Eun-Jeong et al. produced PDMS films with micro-convex structures using PS master molds, employing these films as external light extraction layers to enhance EQE by 42% [27,28]. Additionally, Guixiong Chen et al. used micro lens array molds and applied strain to produce grating-structured micro lens array PDMS films, which enhanced EQE by 2% when used as external light extraction layers [29]. On the other hand, Tae-Woo Lee et al. employed photolithography and Au etching to create line patterns on PDMS for internal light extraction layers [30].

Recent research has highlighted the use of PDMS substrates for nanostructure fabrication through UV or O_2_ plasma treatment [31,32,33]. This method enables the creation of randomly or periodically arranged nanostructures on PDMS films using plasma treatment alone, without the need for advanced techniques such as laser processing, photolithography, nano-transfer processes, or the application of physical forces to the surface. Additionally, by controlling plasma parameters such as power, exposure time, and gas flow rate, the morphology of the nanostructures can be fine-tuned, making this approach highly suitable for innovative OLED light extraction technologies. However, current research remains predominantly focused on the analysis and structural design of these nanostructures, with limited studies addressing their application in OLED light extraction.

In this study, a straightforward and efficient method utilizing oxygen (O_2_) plasma was applied to create periodically wavy-patterned PDMS substrates. This wavy structure, commonly employed in light extraction technologies, was incorporated into OLEDs to increase the critical angle of light. Such an increase reduces the effects of TIR, thereby minimizing losses from waveguide and SPP modes. When exposed to O_2_ plasma, PDMS—a silicon-based material—develops a SiO_x_ layer on its surface. By stretching the PDMS during plasma exposure and then allowing it to revert to its original shape, the formation of a wavy pattern is induced by surface stresses. The impact of the pattern’s period and amplitude on light extraction from OLEDs was meticulously evaluated using finite-difference time-domain (FDTD) simulations, ray-tracing, and power dissipation analyses. The use of O_2_ plasma, commonly employed for substrate cleaning in device fabrication, provides considerably more time and process efficiency than other methods. By adjusting parameters such as gas flow, exposure time, and power, precise control over the formation of wavy patterns with specific amplitudes and periods is achievable.

A green fluorescent OLED, fabricated using the wavy-patterned PDMS substrate, demonstrated enhanced electroluminescence (EL) performance and improved viewing angles. Moreover, FDTD simulations conducted for power dissipation analysis confirmed quantitative enhancements in reducing losses from SPP and waveguide modes, thereby validating the effectiveness of this approach.

## 2. Experimental Section

### 2.1. Wavy-Patterned PDMS Substrate Preparation 

**Figure 1:**
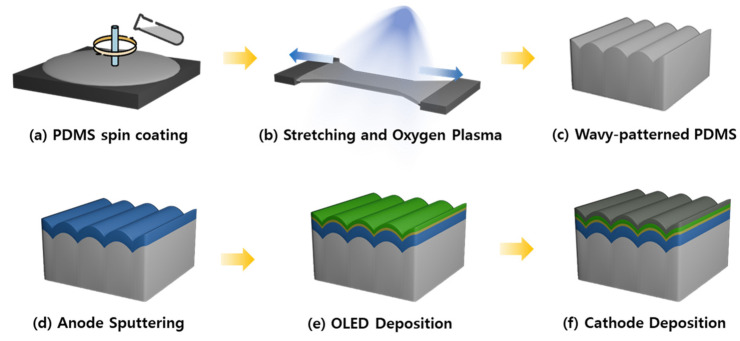
Schematic of the fabrication process for (**a**–**c**) wavy-patterned PDMS substrates and (**d**–**f**) green fluorescent OLEDs.

The fabrication process and structural details of the wavy-patterned PDMS substrate are illustrated in Figure 1. 

First, all PDMS films were applied to an Eagle XG glass substrate (Corning Inc., Corning, NY, USA). The substrate underwent cleaning in an ultrasonic bath using acetone, methanol, and deionized water sequentially, each for 15 min, followed by drying with a N_2_ blower. For the preparation of thin films, UV and O_2_ plasma treatments were conducted for 3 min and 2 min 40 s, respectively. The PDMS elastomer (SYLGARD™ 184, Dow Corning, Midlands, TX, USA) was combined with its curing agent (SYLGARD™ 184 curing agent, Dow Corning, Midlands, TX, USA) at a 10:1 ratio, mixed using a vortex mixer for 5 min, and subsequently degassed in a vacuum chamber for 1 h to eliminate air bubbles. The mixture was then spin-coated onto the pre-cleaned glass substrate at 200 rpm for 60 s and partially cured on a hot plate at 90 °C for 20 min (Figure 1a). 

To generate a wavy pattern, the cured PDMS was removed from the glass substrate and affixed to a custom-made stretching device. Here, strain was applied to elongate the PDMS by 12.5% of its original length. As shown in Figure S1, the wavy pattern was barely visible under low strain (Figure S1a), while higher strain resulted in tearing of the PDMS (Figure S1b). Therefore, the PDMS was stretched to 12.5% of its original length. During this stretched state, O_2_ plasma treatment was executed using a vacuum plasma system (CUTE, Femto Science Inc., Hwaseong, Republic of Korea) (Figure 1b). The treatment conditions were divided into power ranges of 40–100 W, exposure times of 6–12 min, and O_2_ flow rates of 1–100 sccm to observe the resulting changes in amplitude and period. Upon releasing the strain, the PDMS reverted to its original form, displaying a wavy pattern on its surface (Figure 1c).

### 2.2. Fabrication of Green Fluorescent OLED

A transparent anode and an indium zinc oxide (IZO) layer were deposited using a 99.9% pure IZO target from Kojundo Chemical Laboratory Co., Ltd., Sakado, Japan, employing an RF magnetron sputtering system provided by Korea Vacuum Tech, Ltd., Korea. The deposition was carried out at room temperature (25 °C) with a base pressure set at 3 × 10^−6^ Torr. The target-to-substrate distance was maintained at 19 cm, and the substrate rotation speed was 10 rpm to ensure film uniformity. The operating pressure during deposition was 0.8 mTorr, with an argon flow rate of 5.8 sccm, and the power was stabilized at 150 W to match the maximum power density of the target. The thickness of the film was meticulously controlled by adjusting the deposition time, aiming for a final thickness of 200 nm at a deposition rate of 8.34 nm/min (Figure 1d).

Following this, the organic and metallic layers of the OLED were deposited using a thermal evaporator (Korea Vacuum Tech, Ltd., Gimpo, Republic of Korea) under a high vacuum of approximately 10^−7^ Torr onto the glass substrate (Figure 1e). The energy diagram and structure of the OLED are depicted in Figure S2, featuring a layer sequence of 60-nm *N,N*′-bis(naphthalen-1-yl)-*N,N*′-bis(phenyl)-benzidine (NPB) as the hole transport layer (HTL), 80 nm tris(8-hydroxyquinoline) aluminum (Alq_3_) serving both as the EML and the electron transport layer (ETL), 2 nm lithium fluoride (LiF) as the electron injection layer, and 100 nm aluminum as the cathode.

### 2.3. Characterization

The structural features of the wavy-patterned PDMS substrate were analyzed using field-emission scanning electron microscopy (FE-SEM, S-4800, Hitachi, Ltd., Tokyo, Japan). Surface roughness was characterized by atomic force microscopy (AFM, XE-100, Park Systems, Suwon, Republic of Korea). Optical properties, including total transmittance, specific transmittance, and haze of the thin films, were assessed using an ultraviolet–visible–near-infrared (UV–Vis–NIR) spectrophotometer (Cary 5000, Agilent Technologies, Santa Clara, CA, USA). EL values of the OLEDs were measured using a spectroradiometer (PR-670, JADAK, Syracuse, NY, USA) connected to an adjustable voltage source unit (Model 237, Keithley Instruments, Inc., Cleveland, OH, USA) within a controlled environment box. The emitting pixel size was set at 6.25 × 6.25 mm, and the current density was calculated by dividing the measured current by the carrier-injected area of the pixel.

### 2.4. Simulation Analysis

**Figure 2:**
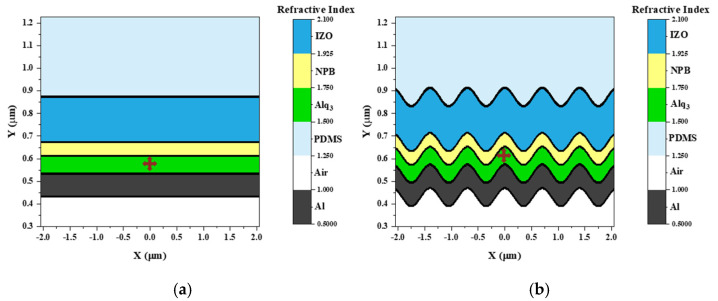
Devices featuring PDMS (**a**) and wavy-patterned OLEDs (**b**) with refractive index monitoring.

To theoretically evaluate the impact of the wave-patterned PDMS substrate on the OLED performance, numerical simulation was conducted using the FDTD method, implemented through Lumerical software (Ansys, Inc., Canonsburg, PA, USA). This technique discretizes the model into unit cells or meshes, facilitating a detailed computational analysis of system behavior. All thin-film layers were stacked along the y-axis, with light emission occurring in the same direction. The material properties (n and k) and thicknesses of the materials were set to mirror the actual experimental conditions. As illustrated in Figure 2, the refractive indices for each layer in both the planar (Figure 2a) and wavy (Figure 2b) configurations were precisely measured using a refractive index monitor. This allowed for an accurate characterization of the optical properties relevant to each layer within the structures. To ensure a detailed representation of the delicate structure and reliable simulation outcomes, computational meshes with a resolution of 2 nm were utilized in the interface region. The electric and magnetic field vector components were calculated by solving Maxwell’s equations in their partial differential forms at each interface. Perfectly matched layers were employed at the simulation boundaries to absorb edge reflections and minimize unwanted light interference, with the anode side designated as the metal boundary. Additionally, a far-field monitor was placed along the x-axis to capture the distribution of light emitted from the OLED through the PDMS. An electric field (E-field) monitor was also strategically positioned to cover the region extending from the OLED device in the direction of light emission. To reduce variations in light extraction efficiency due to the placement of dipoles within the wavy structure, dipoles were strategically positioned at the peaks, troughs, and nodes of the pattern [34]. The isotropic emitter characteristics were assessed by averaging the results from three dipole orientations (x-, y-, and z-polarized), followed by dimensionless normalization. Far-field simulations were carried out over an area exceeding 10 µm to ensure accuracy. The light extraction enhancement (LEE) factor of the wavy-patterned OLEDs was calculated as the ratio of the integrated light intensity measured by the far-field monitor compared to the planar PDMS OLED. Mode efficiency was determined by applying the trapezoidal rule to the calculated power dissipation values. 

To examine the behavior of the light emitted from an OLED into air across different polarization modes, a dispersion relation graph (*k*/2π, *ω*/2πc) was employed [35]. This graph is derived from the far-field graph (*θ*, *λ*) obtained via FDTD simulation, based on Equations (2) and (3):

(2) $$\frac{k}{2\pi }=\frac{sin\theta }{\lambda }\;,$$

(3) $$\frac{\omega }{2\pi c}=\frac{1}{\lambda },\;$$

where *θ* denotes the far-field angle, *ω* denotes the wave frequency, c denotes the speed of light, and *k* denotes the wavevector. By calculating values for the x-axis and y-axis using these equations, a dispersion relation graph plotting *k*/2π against *ω*/2πc can be constructed.

To quantitatively evaluate the light confinement in each optical mode, including radiative, waveguide, and SPP modes, the power dissipation was computed as a function of the normalized in-plane wave vector (u) using MATLAB. Initially, the transfer matrix method [36] was applied to describe relationships based on Maxwell’s equations for multilayer thin films, complemented by the diffraction matrix method [37], which considers diffraction effects from periodic structures. The electric field in real space (x, y coordinates) was subsequently transformed into reciprocal space (*k_x_*, *k_y_* coordinates) through Fourier transformation to calculate the power dissipation density. The reciprocal space coordinates *k_x_* and *k_y_* were then combined to derive form the in-plane wavevector (*k_∥_*), which was normalized to produce the normalized in-plane wavevector (u) [38]. Using this methodology, a power dissipation graph was generated as a function of u, providing insights into optical mode dynamics.

## 3. Results and Discussion

### 3.1. Analysis of Wavy-Patterned PDMS Substrate

**Figure 3:**
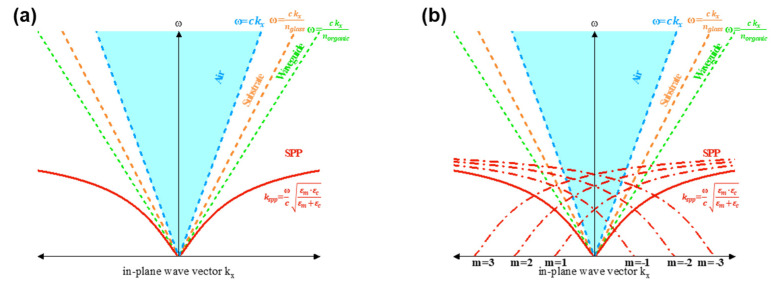
Dispersion relation graphs for (**a**) planar and (**b**) wavy structures.

The behavior of light within OLED structures was investigated using ray tracing and FDTD simulation software. Figure 3 presents the theoretical mode of dispersion of light in both planar and wavy configurations. The turquoise-colored area represents the region where light ultimately exits into the air. Figure 3a illustrates the emission modes of a typical planar OLED, detailing the angular distribution of light across various modes. Within the OLED, losses are incurred through air, substrate, waveguide, and SPP modes at the interface between the organic layer and metal electrode. In standard configurations, losses from substrate and waveguide modes, as well as from SPP modes, result in only about 20% of the generated light being emitted into air, as depicted by the blue region in Figure 3a.

In contrast, Figure 3b demonstrates shows that incorporating applying a wavy structure into the OLED introduces periodicity that satisfies, which enables light emitted from the emissive layer to satisfy the Bragg diffraction condition as follows [39]:

(4) $$k_{x}^{\prime }=k_{0}sin\theta =k_{x}\pm m\cdot \frac{2\pi }{\Lambda }\left(m=\pm 1,\pm 2,\ldots .\right)\;,\;k_{0}=\frac{2\pi }{\lambda },$$

where *k*_0_ represents the wavevector of light in free space, Λ denotes the period of the structure, and mm indicates the diffraction order. This diffraction redirects light from higher-order modes such as *m* = −1, 1, −2, 2… away from SPP and waveguide modes into the air mode. Such redirection significantly enhances light extraction efficiency, as the periodic wavy structure effectively converts trapped light into extractable modes, thus improving the overall optical performance of the OLED [40]. 

**Figure 4:**
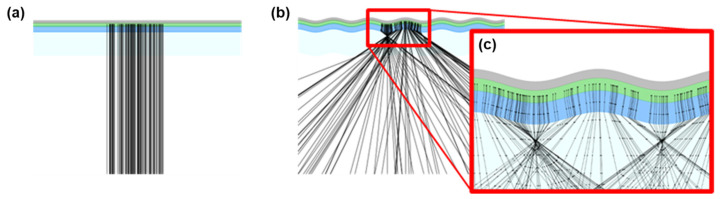
Ray-tracing results showing a (**a**) planar structure, (**b**) wavy structure, and (**c**) magnified view of the diffraction region.

Figure 4 displays the ray-tracing results for OLED emissions on both planar and wavy-patterned PDMS substrates. A ray-tracing simulation was conducted using a custom-developed program that models light propagation paths according to Snell’s law. For the wavy-patterned PDMS substrate, the program calculated tangents to the sinusoidal surface to determine the angles of incidence and reflection, facilitating the implementation of a ray-tracing model to accurately track the paths of light rays.

This methodology enables the prediction and analysis of light behavior within the multilayered thin-film structures of OLEDs. As depicted in Figure 4a,b, the ray-tracing results highlight clear distinctions between the planar and wavy structures. In the planar structure (Figure 4a), light primarily follows straight paths with limited scattering. In contrast, the wavy structure (Figure 4b) induces light refraction and scattering due to its periodic sinusoidal surface, which results in a macroscopic scattering effect that significantly enhances light extraction [12].

### 3.2. FDTD Simulation for Wavy-Patterned OLEDs 

**Figure 5:**
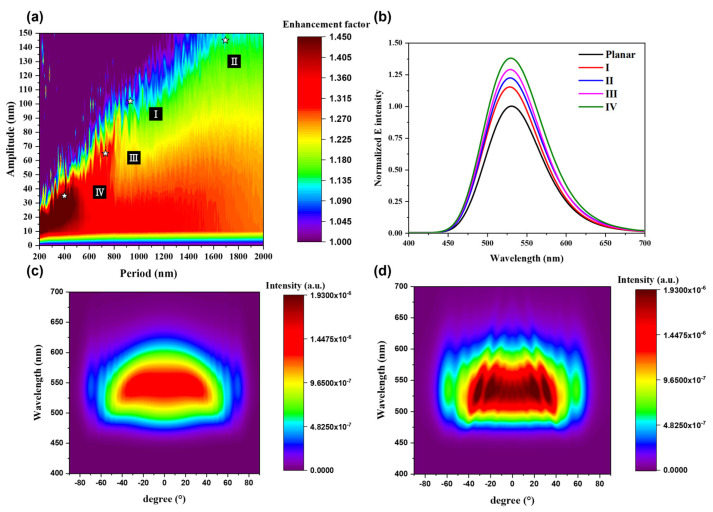
(**a**) Contour plot of the LEE factor as the function of period and amplitude, (**b**) wavelength vs. specific LEE factor graph, and far-field distribution of (**c**) the planar OLED and (**d**) the wavy-patterned substrate with the highest LEE factor (IV).

While ray tracing provides a macroscopic view of light scattering, it is essential to consider microscopic phenomena such as light interference and diffraction. To address this, FDTD simulations were performed to analyze far-field properties. Figure 5 displays the results, showing the LEE and far-field distributions as functions of the amplitude and period variations of the wavy structure.

Initially, a planar reference device was configured by optimizing the electrode (Figure S3) of the OLED. The optimization process involved varying the period and amplitude of the wavy structure. As illustrated in Figure 5a, specific regions exhibited enhanced light extraction. Moreover, an increase in the period-to-amplitude ratio was found to enhance transverse electric (TE)-mode light coupling with SPPs, leading to energy losses and a reduction in the enhancement factor [41]. Conversely, when the ratio decreased, diffraction effects were minimal, resulting in a lower enhancement factor. These observations led to the experimental optimization of period and amplitude parameters to achieve structures corresponding to regions with the highest enhancement factors [41].

To approximately quantify the improvement in light extraction and understand the emission characteristics, the intensity of the OLEDs with enhancement factors of 1.15 (I), 1.2 (II), 1.3 (III), and 1.4 (IV) at a wavelength of 525 nm was plotted (Figure 5b). Figure 5c,d compare the device with the highest enhancement to a PDMS-based device. The findings showed an increase in forward intensity (0°) and maintained high intensity up to 60°, indicating an improved viewing angle. These simulations demonstrated that the periodic wavy structure effectively induces diffraction and facilitates the extraction of light in the SPPs and waveguide modes, thereby enhancing the intensity of light emitted from the OLED.

### 3.3. Surface Morphology of Wavy-Patterned PDMS Substrate

**Figure 6:**
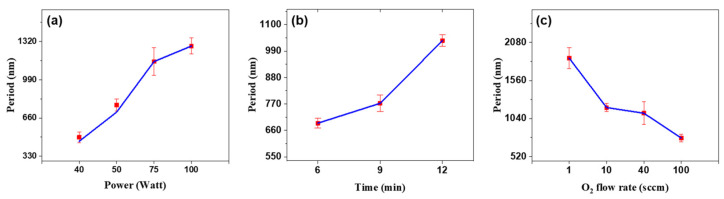
Changes in the period based on (**a**) O_2_ plasma power (40–100 W), (**b**) exposure time (6–12 min), and (**c**) O_2_ flow rate (1–100 sccm). Error bars represent standard deviations from at least three SEM measurements across distinct regions.

Efficient light extraction from green OLEDs necessitates the design of structures with specific amplitudes and periods that facilitate light diffraction within certain wavelength ranges. These parameters can be finely tuned using O_2_ plasma treatment settings. Figure 6 illustrates the effect of varying the power (time = 9 min, O_2_ flow rate = 100 sccm), exposure time (power = 50 W, O_2_ flow rate = 100 sccm), and O_2_ flow rate (power = 50 W, time = 9 min) during plasma treatment on the periods of the wavy patterns. Figure 6a,b demonstrate that both the period and amplitude increase with higher power and longer treatment duration, which is a result of increased oxidation on the PDMS surface due to higher plasma energy. Stress relaxation and redistribution in double-layered thin films, which possess differing stretching or thermal expansion coefficients, are typically employed to generate wavy structures [42,43]. As the plasma power increases, the thickness of the SiO_x_ layer also increases, resulting in a pronounced stress mismatch between SiO_x_ layer and PDMS. This enhanced mismatch leads to an increase in both the period and amplitude of the wavy structures.

Moreover, Figure 6c reveals that an increase in O_2_ flow rate leads to a decrease in the period. This effect is attributed to the rise in chamber pressure caused by the higher O_2_ influx, which shortens the mean free path of the O_2_ molecules [33,43], reducing the energy impacting the PDMS surface [44]. This analysis confirms the dependency of the period on the O_2_ plasma conditions and facilitated structural optimization, as validated by SEM and AFM analysis. 

When comparing experimentally obtained periods with simulation results, it was found that a period below 800 nm is necessary to enhance light extraction in green fluorescence OLEDs. However, as depicted in the SEM image in Figure S4, periods below 700 nm resulted in significant uniformity degradation and the development of cracks within the wavy structures. To address this issue, an optimal period of approximately 700 nm was achieved using 50 W plasma power and a treatment duration of 9 min. Since the O_2_ flow rate had a minimal impact on crack formation, it was varied independently to assess the optical characteristics and OLED performance in terms of diffraction efficiency [33].

**Figure 7:**
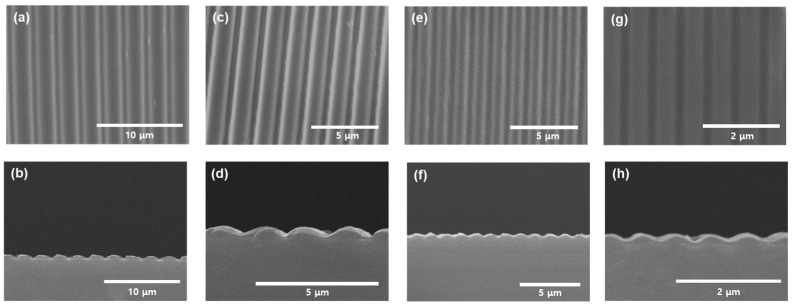
Top- and cross-sectional-view SEM images of (**a**,**b**) S-1, (**c**,**d**) S-10, (**e**,**f**) S-40, and (**g**,**h**) S-100.

Figure 7 presents the SEM top and cross-sectional views of wavy-patterned PDMS substrates fabricated under varying O_2_ plasma flow rates. Here, S-x refers to samples prepared under process conditions with an O_2_ flow rate of x. As the intensity of the O_2_ plasma treatment decreases, the SEM top-view images (Figure 7a,c,e,g) show a reduction in the period of the wavy structures. As summarized in Table 1, the period increased from 730 nm to 1700 nm over a 5 μm range. Additionally, the cross-sectional SEM images (Figure 7b,d,f,h) reveal a decrease in amplitude. 

**Figure 8:**
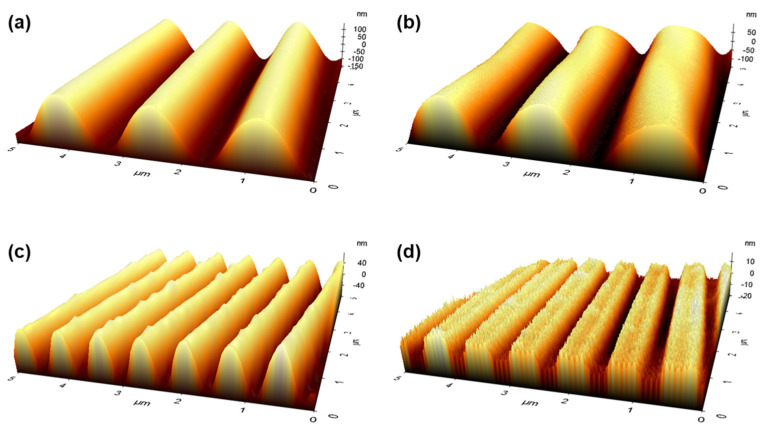
AFM images of wavy-patterned structures: (**a**) S-1, (**b**) S-1/IZO, (**c**) S-100, and (**d**) S-100/IZO.

To obtain more precise measurements of the amplitude and surface roughness after the application of the IZO electrode, further analyses were conducted using AFM. Figure 8 shows the AFM images of S-1 and S-100 with and without IZO, respectively. As summarized in Table 1, the amplitude decreased from 150 nm to 72 nm over a 5 μm range. Additionally, the Fourier transform of these structures, depicted in Figure S5, indicates two high-intensity points, characteristic of periodic wavy structures. These findings confirm that the fabricated structures align well with those modeled in simulations [45]. However, this analysis primarily focuses on optical properties, and it should be noted that the amplitude also influences the sheet resistance of materials. Current OLED IZO electrodes incorporate a 200 nm layer, and their surface characteristics are depicted in Figure 7c,f. Compared to the uncoated PDMS, the amplitude decreased by 12% and 75% to 128 nm and 18 nm, respectively, while surface roughness persisted. The sheet resistance values are detailed in Table 1 and Figure S6. For planar PDMS substrates, a sheet resistance of 25.4 Ω/sq was measured. In wavy-patterned PDMS substrates, sheet resistance increased as the period and amplitude decreased, with more pronounced variations observed. Notably, although the sheet resistance increased to 29.74 Ω/sq (~16% increase) at 1 sccm, this level remained suitable for OLED electrode applications. The 100 sccm wavy structure, which showed high optical efficiency in simulations, demonstrated a sheet resistance of 25.55 Ω/sq, nearly equivalent to that of planar substrates, indicating that the wavy structure does not have a detrimental impact on the electrical performance of the OLED electrodes.

### 3.4. Optical Properties of the Wavy-Patterned PDMS Substrate

**Figure 9:**
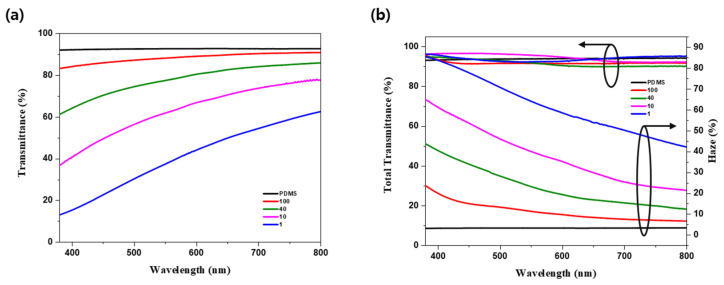
Optical characteristics of wavy structures on PDMS substrates at varying O_2_ flow rates: (**a**) optical transmittance and (**b**) total optical transmittance and haze as functions of visible wavelength.

Figure 9 illustrates the transmittance, total transmittance, and haze of PDMS samples modified by varying O_2_ flow rates. Owing to the different periods and amplitudes, diffraction effects were anticipated. To quantify these diffraction impacts, the haze characteristics were analyzed using the following equation [46,47]:

(5) $$haze=\frac{T_{diffuse}}{T_{total}}\times 100,$$

where *T_total_* represents the total transmittance measured with an integrating sphere, which captures the light transmitted at all angles. *T_diffuse_* measures the scattered light while excluding the direct transmission (*T_direct_*). Consequently, haze is defined as the ratio of *T_diffuse_* to *T_total_*. The measured transmittance, total transmittance, and calculated haze values are listed in Table 2.

Compared to the planar PDMS samples, the transmittance decreased while the haze increased across all modified samples, which is attributed to enhanced light diffraction. This effect was visually evident, as illustrated in Figure S7. For instance, both the S-100 sample, subjected to the lowest plasma power, and the S-1 sample, subjected to the highest power, demonstrated a total transmittance exceeding 90%, indicating that the PDMS does not absorb light but rather scatters and diffracts it. Specifically, transmittance decreased from 92.62% to 87.71% and 33.97% for the S-100 and S-1 samples, respectively, while haze increased from 3.36% to 12.37% and 67.31%, respectively. These changes suggest that the periodic structures and increased surface amplitude contribute significantly to diffraction. Furthermore, as periodicity and amplitude increased, transmittance decreased progressively and haze increased significantly, confirming an enhanced degree of light diffraction. These observations provide a foundation for further investigation into how varying diffraction levels affect the emission characteristics of OLEDs.

### 3.5. OLED Performance

**Figure 10:**
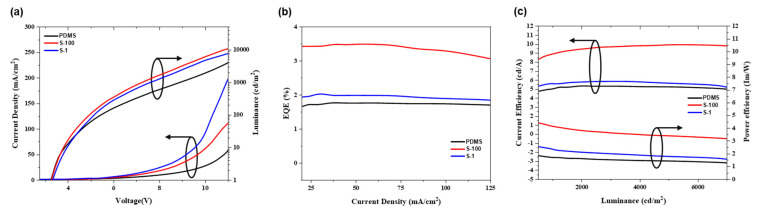
Comparison of EL characteristics of the PDMS and wavy-patterned devices (S-100 and S-1) in green fluorescent OLEDs: (**a**) current density–voltage–luminance characteristics, (**b**) EQE–current density, (**c**) CE–luminance–PE characteristics.

Figure 10 and Table 3 detail the current–voltage–luminance (J–V–L) characteristics, EQE, current efficiency/power efficiency (CE/PE), and peak wavelength of the wave-patterned OLED structures (S-100 and S-1) and the reference device (PDMS). Figure 10a displays the J–V–L characteristics of each device. The maximum luminance achieved was 6397 cd/m^2^ for the PDMS, 11,550 cd/m^2^ for S-1, and 9289 cd/m^2^ for S-100. This is also consistent with the LEE factor graph in Figure 5, which shows that the S-100 device (point III in Figure 5b) exhibits higher intensity compared to the S-1 device (point II in Figure 5b). These results represent luminance increases of approximately 80% and 45% for S-1 and S-100, respectively, compared to that of the PDMS device. The current density increases in the order PDMS < S-100 < S-1. This trend is attributed to the increased amplitude of the wave pattern, which caused the organic layers in the sloped regions to thin compared to those at the nodes and antinodes. Consequently, a shorter charge transport path in these regions resulted in a stronger electric field, leading to a higher current density [47,48].

Despite the surface roughness introduced by the wavy pattern, the turn-on voltage of the devices remained nearly unchanged [49]. Figure 10b presents the EQE of each device. The maximum EQE values for S-100 and S-1 were measured at 3.5% and 2.03%, respectively, representing 97% and 15% enhancements compared to the planar PDMS device. Figure 10c displays the CE and PE of the devices, comparing the PDMS and S-100 devices. The CE increased from 5.35% to 9.95%, representing an enhancement of approximately 86%. Similarly, the PE improved from 2.28% to 4.64%, showing an increase of approximately 104%. These results demonstrate the effectiveness of the wavy structure in targeting the 525 nm wavelength emitted from OLEDs, facilitating the diffraction and extraction of light that would otherwise be trapped by TIR, waveguide modes, and SPP losses.

**Figure 11:**
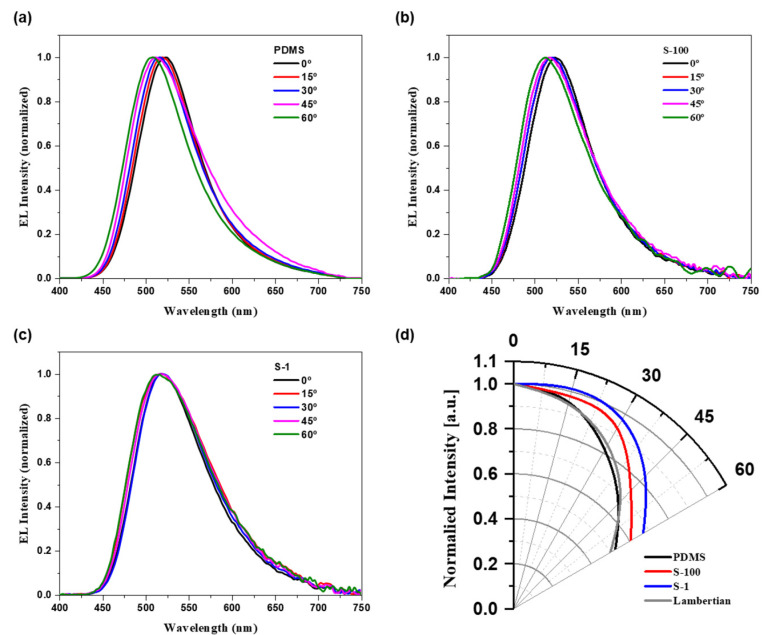
Normalized EL spectra at visible wavelengths from 0° to 60° for the (**a**) PDMS, (**b**) S-100, and (**c**) S-1 devices. (**d**) Normalized angular luminance distributions of the PDMS, S-100, and S-1 devices compared with the Lambertian distribution. All EL characteristics were measured at a current density of 50 mA/cm^2^ in green fluorescent OLEDs.

**Figure 12:**
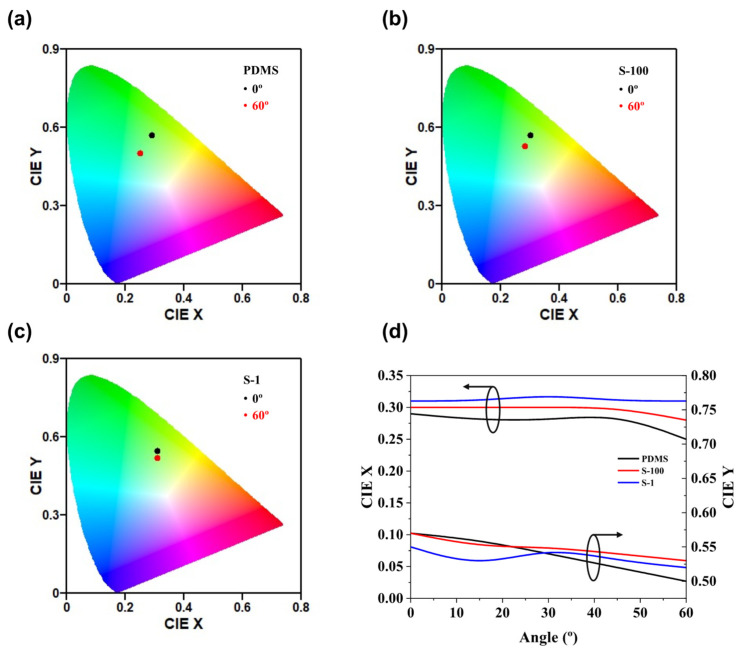
CIE coordinates at 0° to 60° for the (**a**) PDMS, (**b**) S-100, and (**c**) S-1 devices. (**d**) CIE coordinates as a function of the viewing angle from 0° to 60° for each device. All EL characteristics were measured at a current density of 50 mA/cm^2^ in green fluorescent OLEDs.

Figure 11 and Figure 12 and Table 4 illustrate the color shifts of each device at viewing angles ranging from 0° to 60°. Figure 11 details the changes in peak wavelength and Lambertian emission characteristics. The PDMS device exhibited a hypsochromic shift of approximately 16 nm, while devices with structured substrates showed reduced hypsochromic shifts as the haze increased, indicating enhanced diffraction. Specifically, the peak wavelength shifts for S-100, and S-1 were 10, and 6 nm, respectively. Furthermore, while the PDMS device closely adhered to the Lambertian distribution due to its bottom-emission structure [50], devices with structured substrates demonstrated enhanced light extraction at all angles, owing to diffraction. This resulted in a broader viewing angle compared to both the Lambertian model and the PDMS device, highlighting the benefits of structured substrates in broadening the angular response and enhancing the visual performance of OLEDs.

Figure 12 illustrates the chromaticity shifts for all devices. Both S-100 and S-1 demonstrated smaller chromaticity shifts compared to the PDMS device as the viewing angle increased from 0° to 60°. The CIE X and Y coordinates show a decreasing trend in the order of PDMS, S-100, and S-1. At a viewing angle of 60°, the chromaticity shifts for the PDMS, S-100, and S-1 devices were 0.081, 0.044, and 0.030, respectively. These findings indicate that devices with structured substrates exhibit markedly improved chromaticity stability.

### 3.6. Analysis of SPP Mode

SPPs are electromagnetic waves that arise from the interaction between electron plasma and incident electromagnetic waves at the metal–dielectric interface. These waves propagate along the interface and exhibit resonant damping characteristics. SPPs are predominantly observed in the direction perpendicular to the metal–dielectric interface due to their electric field components, making them detectable solely in the transverse magnetic (TM) mode. In contrast, in the TE mode, where the electric field is aligned parallel to the interface, the conditions necessary for inducing surface plasmons are not met [51]. Therefore, SPPs are relevant exclusively in the TM mode, which provides a critical foundation for the design and analysis of optical components in OLED devices. 

**Figure 13:**
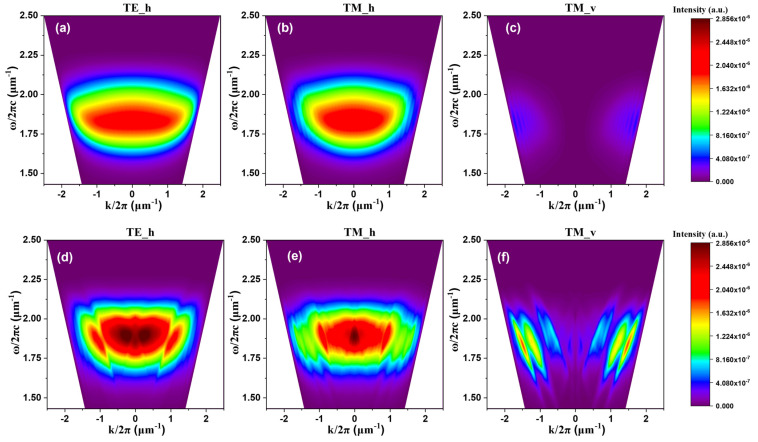
Dispersion relations in (**a**–**c**) planar and (**d**–**f**) wavy structures: (**a**,**d**) horizontal TE modes, (**b**,**e**) horizontal TM modes, and (**c**,**f**) vertical TM modes.

Figure 13 presents the dispersion relationships of OLEDs on both planar and wavy-patterned PDMS substrates, as determined by FDTD simulations for horizontal TE, horizontal TM, and vertical TM polarization modes. These dispersion relations quantitatively depict the interactions between wave frequency and wave vector, facilitating an analysis of the effects of material properties and structural design on device performance. Furthermore, they enable a macroscopic evaluation of how effectively light trapped in various modes is extracted through Bragg scattering, as categorized by polarization modes.

The dispersion relations reveal that across horizontal TE, horizontal TM, and vertical TM modes, the wavy structure exhibited higher intensities compared to the planar structure. These relations displayed linear patterns, indicating the extraction of previously dissipated light through Bragg diffraction at specific diffraction orders [35]. In Figure 13a, the horizontal TE mode is characterized by light intensity arising primarily from the waveguide mode. This is due to the requirement of SPPs for the electric field to align perpendicular to the metal interface (a condition unmet in the horizontal TE mode), thus facilitating analysis of waveguide mode suppression.

Figure 13b,c illustrate simulation results for the other two polarization modes: horizontal TM and vertical TM. In these modes, both the waveguide and SPPs are present; however, new intensities compared to the planar structure confirm that Bragg diffraction enhances light extraction across all modes. 

Figure S8 presents the E-field monitoring results for each of the three modes at a wavelength of 525 nm, visually depicting the distribution of light across each layer of the OLED [34]. The analysis indicates that in the planar structure, light is predominantly confined within the bottom Al layer. However, the introduction of the wavy structure facilitates some light extraction into the air, thereby reducing the SPPs at the Al–organic interface.

**Figure 14:**
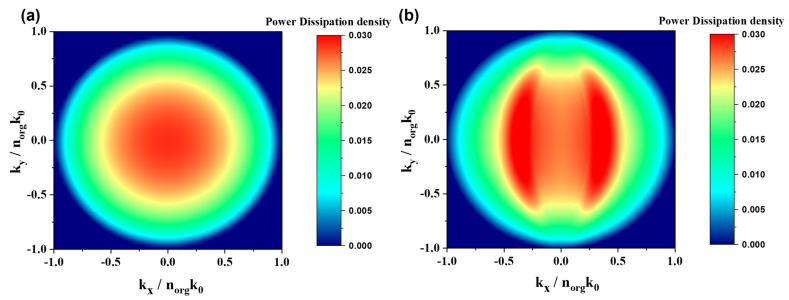
Power dissipation density in the Fourier space for (**a**) planar and (**b**) wavy-patterned devices at λ = 525 nm.

Owing to limitations in the FDTD simulation for quantitatively analyzing the SPP mode, the characteristics of the modes were precisely analyzed through power dissipation calculations performed using MATLAB. Figure 14 illustrates the reciprocal space power dissipation density of OLEDs with both planar and wavy structures at 525 nm. For the planar structure (Figure 14a), the intensity was highest at the center and decreased uniformly in all directions. In contrast, the wavy-patterned structure (Figure 14b) demonstrated an increased intensity, attributable to the formation of patterns that facilitate the shift and extraction of light trapped in the waveguide and SPP modes through Bragg diffraction.

**Figure 15:**
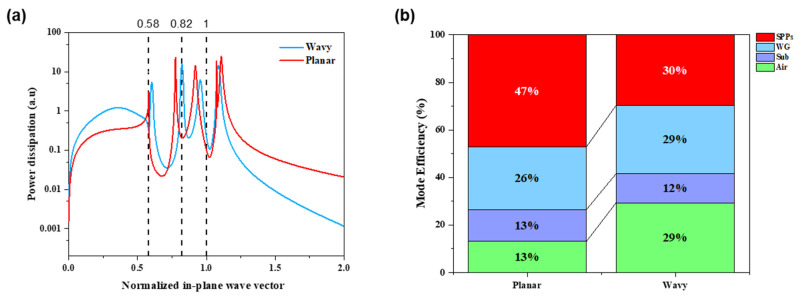
(**a**) Power dissipation spectra in the normalized in-plane wavevector and (**b**) distribution of all optical loss channels at λ = 525 nm for PDMS (red line) and wavy-patterned (blue line) devices.

Figure 15 illustrates the distribution of power dissipation and optical loss at a wavelength of 525 nm. As depicted in Figure 15a, distinct regions of the in-plane wave vector characterize each power dissipation mode. For this wavelength, the normalized in-plane wavevector (u) categorizes the modes as follows. When u < 0.58 (n_air_/n_org_), the light corresponds to the air mode; when 0.58 < u < 0.82 (n_substrate_/n_org_), the light corresponds to the substrate mode; when 0.82 < u < 1, the light is in the waveguide mode; and when u > 1, the light is confined within the SPP mode [52]. 

Analysis of the graph reveals that for u > 1, power dissipation in the wavy structure decreases more steeply than in the planar structure as u increases, indicating a reduction in losses due to SPPs at the interface between the metal and organic layers. Figure 15b presents the mode efficiency, calculated using the trapezoidal rule. The SPP mode in the wavy structure showed a 17% reduction compared to the planar structure, while the substrate mode remained nearly unchanged. The waveguide mode experienced a 3% increase in the wavy structure relative to the planar structure, suggesting that some light extracted from the SPP mode was redirected into the waveguide mode. Furthermore, the air mode demonstrated a 16% increase in efficiency in the wavy-patterned structure compared to the planar structure.

These results, corroborated by FDTD simulations, confirm that light is being effectively extracted from both the waveguide and SPP modes. This optical computation provides a substantial explanation for the observed enhancements in the EL characteristics and viewing angle properties of the OLEDs, illustrating the impact of structural modifications on optical performance. 

## 4. Conclusions

This study demonstrated the enhancement of light extraction efficiency and viewing angle characteristics in bottom-emitting green OLEDs through the use of a wavy-patterned PDMS substrate. By tuning the O_2_ plasma parameters (flow rate, power, and time) and mechanical strain, it was possible to precisely control the amplitude and period of the wave patterns. The introduction of these wave patterns facilitated diffraction and scattering effects, enabling the adjustment of optical properties to achieve varying haze levels while maintaining a high total transmittance exceeding 90%. Employing a wavy-patterned PDMS substrate which mirrored the haze levels anticipated from FDTD simulations significantly enhanced the light extraction efficiency of the green fluorescent OLEDs. This enhancement resulted in an 80% increase in EL performance and a 97% improvement in EQE compared to those of the PDMS device. Additionally, at a luminance of 6000 cd/m^2^, both the CE and PE increased substantially by 93% and 145%, respectively. The modifications also improved the viewing angle characteristics, reducing the CIE coordinate shifts from 0.081 to 0.044 and the peak wavelength shifts from 16 nm to 10 nm as the viewing angle increased from 0° to 60°. The superior EL performance of wave-patterned OLEDs underscores the potential of this approach as a next-generation nanostructure technology. This study establishes a foundation for further research into O_2_ plasma nanopatterning techniques, aiming to address the existing limitations of OLED technologies and pave the way for the development of more efficient and durable devices.

## Figures and Tables

**Table 1:** Amplitude, period, and sheet resistance of wavy-patterned PDMS substrates at varying O_2_ flow rates.

	PDMS	S-100	S-40	S-10	S-1
Amplitude (nm)	-	72	102	120	150
Period (nm)	-	730	930	980	1700
Sheet resistance (Ω/□)	25.4	25.55	26.26	27.88	29.74

**Table 2:** Optical properties of wavy-patterned PDMS substrates at different O_2_ flow rates, measured at a wavelength of 525 nm.

λ = 525 nm	PDMS	S-100	S-40	S-10	S-1
Transmittance (%)	92.62	87.71	76.07	59.37	33.97
Total transmittance (%)	93.80	91.58	92.47	96.03	92.32
Haze (%)	3.36	12.37	25.74	42.88	67.31

**Table 3:** Summary of EL characteristics for PDMS and wavy-patterned devices (S-100 and S-1) in green fluorescent OLEDs.

OLED		PDMS	S-100	S-1
EQE (%)	Maximum	1.77	3.50	2.03
	J = 150 mA/cm^2^	1.62	2.65	1.72
Current efficiency (cd/A)	Maximum	5.35	9.95	5.86
	L = 6000 cd/m^2^	5.14	9.91	5.54
Power efficiency (lm/W)	Maximum	2.28	4.64	2.90
	L = 6000 cd/m^2^	1.34	3.28	1.70
Maximum luminance (cd/m^2^)		6397	11550	9289
Turn-on voltage (V)		3.26	3.26	3.30

**Table 4:** Shifted values of EL spectra and CIE coordinates as the viewing angle varied from 0° to 60° for the PDMS, S-100, and S-1 devices in green fluorescent OLEDs.

Green OLEDs	PDMS	S-100	S-1
Peak wavelength shift (nm)	16	10	6
∆ (x,y)	0.081	0.044	0.030

## Data Availability

The raw data supporting the conclusions of this article will be made available by the authors on request.

## Supplementary Materials

The following supporting information can be downloaded at: https://www.mdpi.com/article/10.3390/nano15030198/s1. This includes SEM images of wavy-patterned PDMS under lower strain and photographs of PDMS under higher strain (Figure S1), an energy-level diagram of the green fluorescent OLEDs (Figure S2), optimization of IZO thickness (Figure S3), SEM images of a wavy-patterned PDMS with periods below 700 nm (Figure S4), fast Fourier transform analysis of a wavy-patterned PDMS (Figure S5), sheet resistance measurements of a wavy-patterned PDMS (Figure S6), photographs of PDMS substrates (Figure S7), and steady-state electric field distributions of a wavy-patterned PDMS as determined by FDTD simulation (Figure S8).

## Abbreviations

The following abbreviations are used in this manuscript:

AFM	atomic force microscopy
PDMS	polydimethylsiloxane
OLED	organic light-emitting diodes
SPPs	surface plasmon polaritons
EML	emission layer
ETL	electron transport layer
HTL	hole transport layer
FDTD	finite-difference time-domain
EL	electroluminescence
EQE	external quantum efficiency
CE	current efficiency
PE	power efficiency
SEM	scanning electron microscopy
