# Supplementary material for: Enhancement of Light Extraction Efficiency Using Wavy-Patterned PDMS Substrates
Source: Nanomaterials (Basel). 2025 Jan 27;15(3):198. doi: 10.3390/nano15030198 (PMC11821116; doi:10.3390/nano15030198)
Supplement: Supplementary file 1 [file nanomaterials-15-00198-s001.zip › nanomaterials-3433150-supplementary.pdf]

Supporting Information

Enhancement of Light Extraction Efficiency Using Wavy-Patterned PDMS Substrates

Jian Cheng Bi [1,†], Kyo-Cheol Kang [1,†], Jun-Young Park [1], Junbeom Song [1], Ji-Sung Lee [1], Hyejung Lim [1], Young Wook Park [2,*] and Byeong-Kwon Ju [1,*]

[1] Display and Nanosensor Laboratory, Department of Electrical Engineering, Korea University, Seoul 02841, Republic of Korea; vlfrkatjd@korea.ac.kr (J.C.B.); kgc430@korea.ac.kr (K.-C.K.); mrjoon123@korea.ac.kr (J.-Y.P.); jnh04107@korea.ac.kr (J.S.); js103412@korea.ac.kr (J.-S.L.); hyejung07@korea.ac.kr (H.L.)

[2] Department of Semiconductor and Display Engineering, Sun Moon University, Asan-si 31460, Republic of Korea

* Correspondence: zerook@sunmoon.ac.kr (Y.W.P.); bkju@korea.ac.kr (B.-K.J.)

† These authors contributed equally to this work.

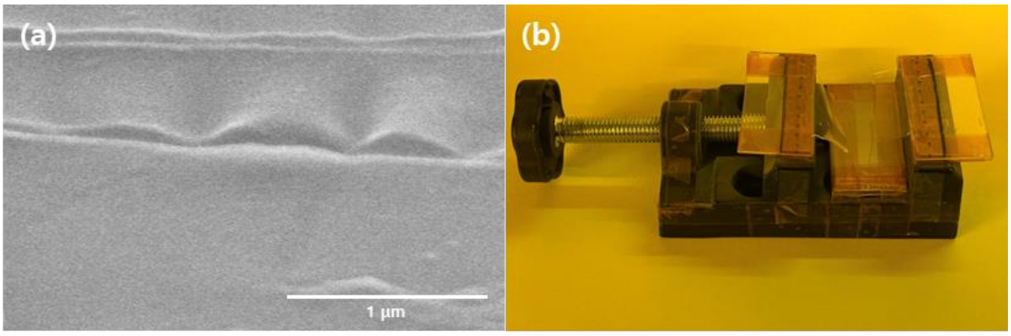

Figure S1. (**a**) SEM images of wavy-patterned PDMS under lower strain and (**b**) photograph of PDMS under higher strain.

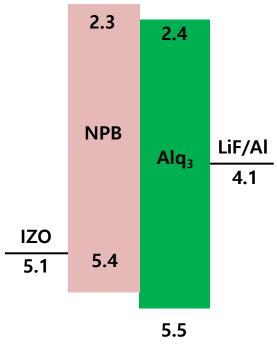

Figure S2. Energy level diagram of the green fluorescence OLEDs employed in this study.

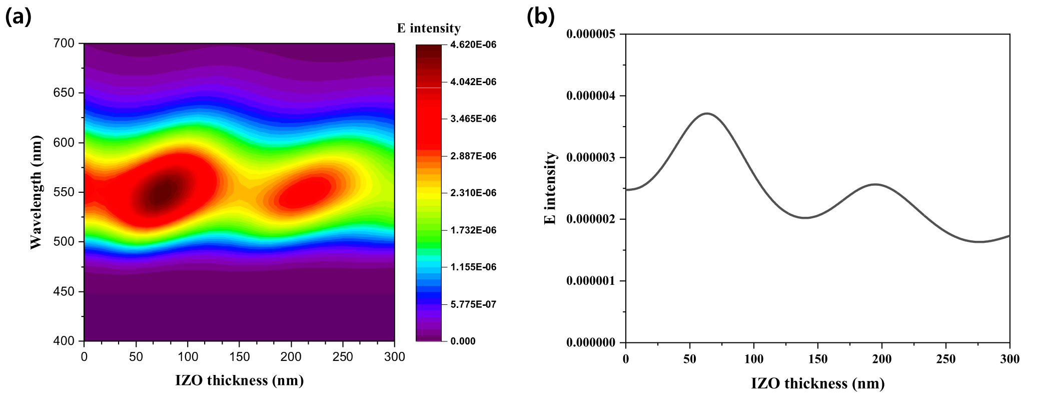

Figure S3. IZO thickness optimization: IZO thickness (**a**) sweep and (**b**) E intensity at 525 nm wavelength.

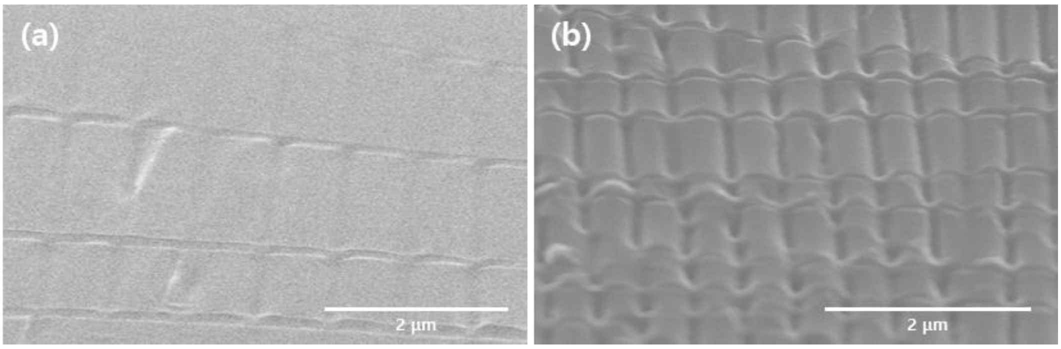

Figure S4. SEM images of wavy-patterned PDMS with periods below 700 nm: (**a**) 6 min condition from Figure 6b, (**b**) 40 W condition from Figure 6a.

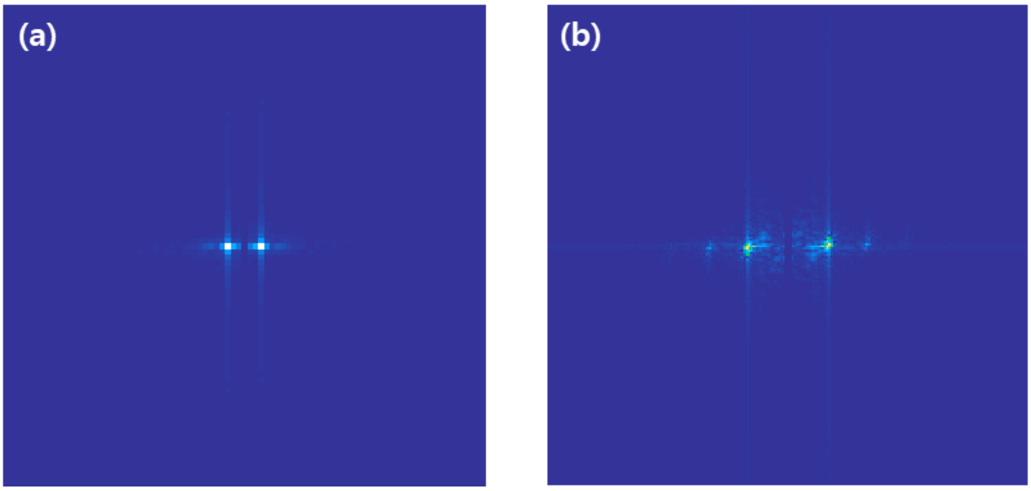

Figure S5. Fast Fourier Transform of wavy-patterned PDMS: (**a**) S-1 and (**b**) S-100.

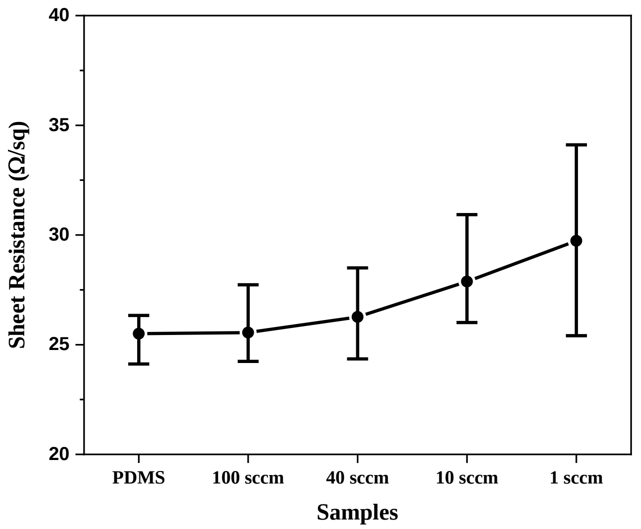

Figure S6. Sheet resistance of wavy-patterned PDMS with different O_2 flow rate.

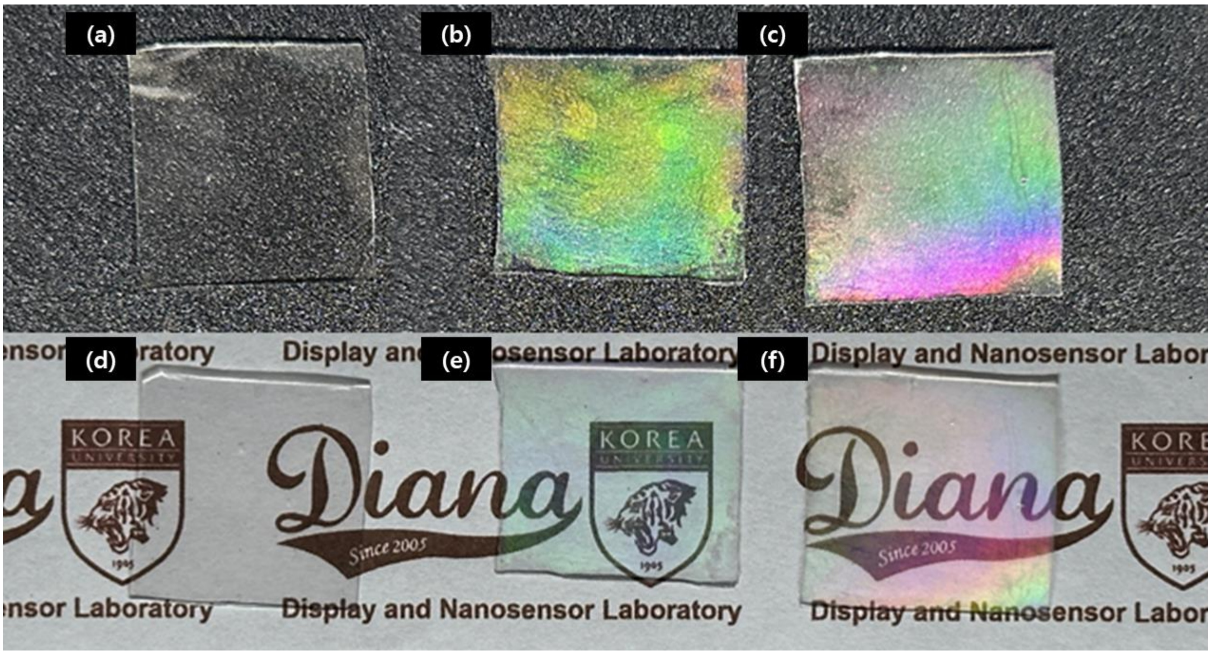

Figure S7. Photograph of wave-patterned PDMS substrate in (**a**–**c**) dark background and (**d**–**f**) white background: (**a**,**d**) planar, (**b**,**e**) S-1 and (**c**,**f**) S-100.

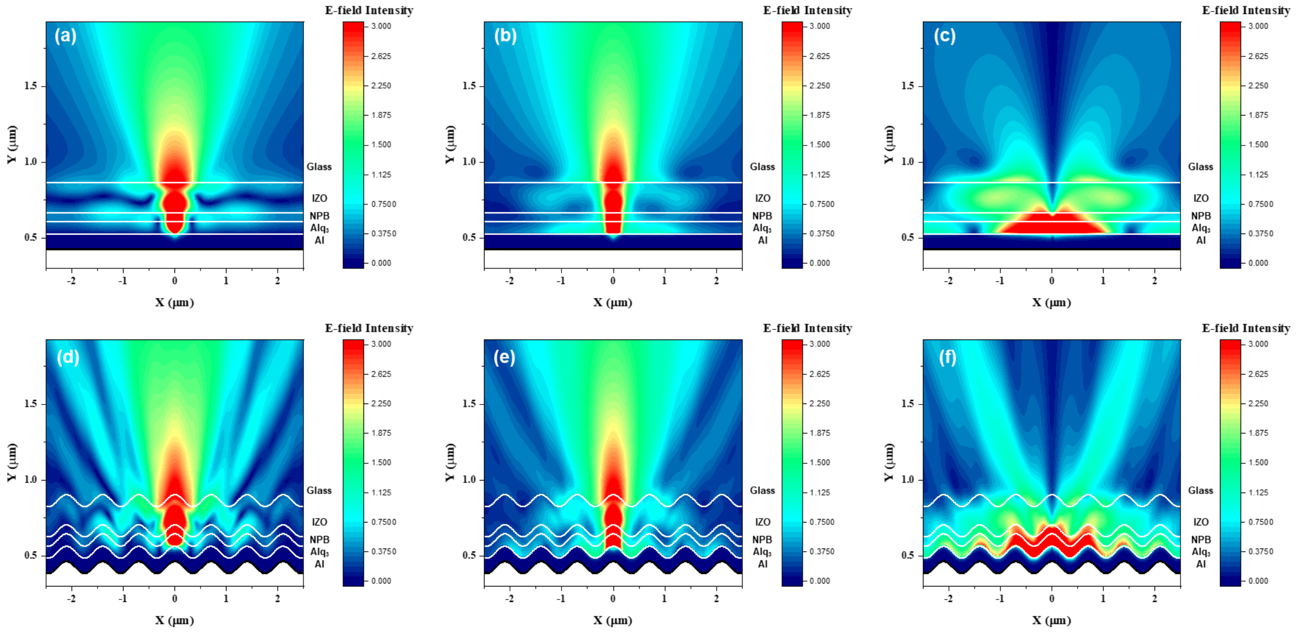

Figure S8. Steady-state electric field distributions of dipoles at λ = 525 nm in (**a**–**c**) planar and (**d**–**f**) wavy structure: (**a**,**d**) Horizontal TE modes, (**b**,**e**) Horizontal TM modes, and (**c**,**f**) Vertical TM modes.
